# Database fingerprint (DFP): an approach to represent molecular databases

**DOI:** 10.1186/s13321-017-0195-1

**Published:** 2017-02-06

**Authors:** Eli Fernández-de Gortari, César R. García-Jacas, Karina Martinez-Mayorga, José L. Medina-Franco

**Affiliations:** 1 Departamento de Farmacia, Facultad de Química, Universidad Nacional Autónoma de México, Avenida Universidad 3000, 04510 Mexico City, Mexico; 2 Instituto de Química, Universidad Nacional Autónoma de México, Avenida Universidad 3000, 04510 Mexico City, Mexico; 3 Escuela de Sistemas y Computación, Pontificia Universidad Católica del Ecuador Sede Esmeraldas (PUCESE), Esmeraldas, Ecuador

**Keywords:** Diversity, Information content, Molecular fingerprints, Similarity, Shannon entropy

## Abstract

**Background:**

Molecular fingerprints are widely used in several areas of chemoinformatics including diversity analysis and similarity searching. The fingerprint-based analysis of chemical libraries, in particular of large collections, usually requires the molecular representation of each compound in the library that may lead to issues of storage space and redundant calculations. In fact, information redundancy is inherent to the data, resulting on binary digit positions in the fingerprint without significant information.

**Results:**

Herein is proposed a general approach to represent an entire compound library with a single binary fingerprint. The development of the database fingerprint (DFP) is illustrated first using a short fingerprint (MACCS keys) for 10 data sets of general interest in chemistry. The application of the DFP is further shown with PubChem fingerprints for the data sets used in the primary example but with a larger number of compounds, up to 25,000 molecules. The performance of DFP were studied through differential Shannon entropy, k-mean clustering, and DFP/Tanimoto similarity.

**Conclusions:**

The DFP is designed to capture key information of the compound collection and can be used to compare and assess the diversity of molecular libraries. This Preliminary Communication shows the potential of the novel fingerprint to conduct inter-library relationships. A major future goal is to apply the DFP for virtual screening and developing DFP for other data sets based on several different type of fingerprints.

**Graphical Abstract:**
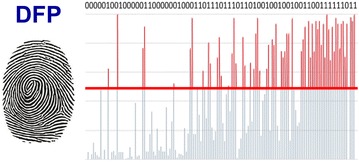
Database fingerprint captures the key information of molecular databases to perform chemical space characterization and virtual screening

**Electronic supplementary material:**

The online version of this article (doi:10.1186/s13321-017-0195-1) contains supplementary material, which is available to authorized users.

## Background

The concept of molecular similarity is commonly used in different areas of chemistry including drug discovery. This is because one of the core paradigms in drug design is that similar compounds share similar properties. A number of molecular representations and similarity coefficients have been proposed [1] to quantify the molecular similarity between single molecular structures and compound libraries.

**Fig. 1:**
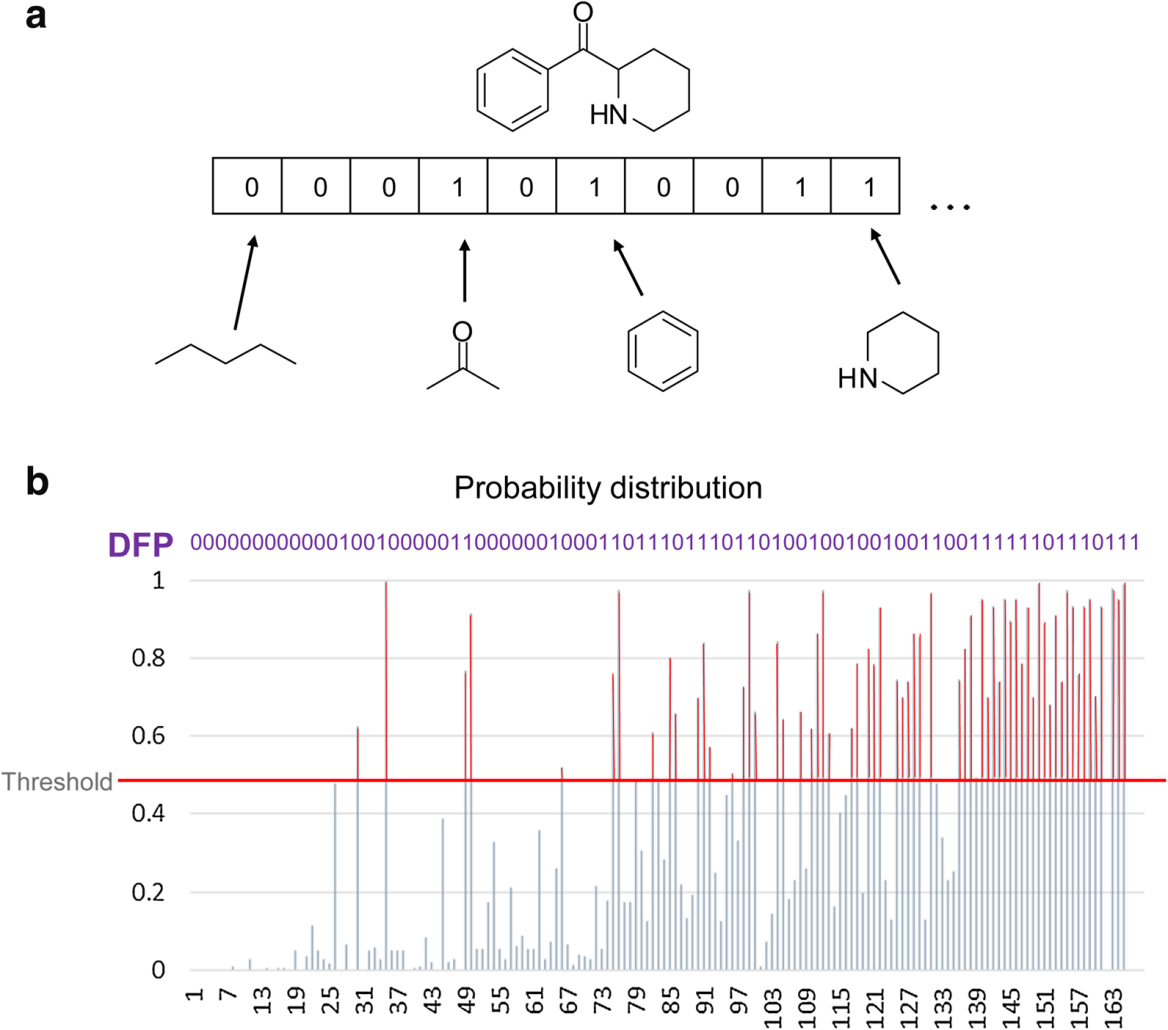
**a** Schematic representation of a binary and dictionary-based molecular fingerprint. **b** Schematic representation of a database fingerprint (DFP)

In chemoinformatics, molecular fingerprints are one of the most common representations of chemical structures. Representations of this type are simplifications of the chemical information contained in any chemical entity through binary vectors. Figure 1a illustrates a schematic representation of a binary fingerprint representation of a chemical structure. Each position in the vector indicates the absence (0) or presence (1) of features predetermined in the design of the fingerprint. For instance, binary vectors developed thus far are the Molecular ACCess System (MACCS) keys [2] and PubChem fingerprints. Despite the fact binary fingerprints lacks of accuracy, they have the advantage of increasing calculation speed and reducing storage space. These features, combined with broad applicability for several years have made molecular fingerprints one of the standard representations to measure molecular diversity among several other applications. However, since the amount of information stored in molecular databases is increasing constantly, there is a need to generate simplifications of the molecular representation of compound databases to open new approaches to studies of the chemical space, optimize the storage and enhance the speed of computations.

The goal of this work was to introduce a new binary fingerprint that encodes the main features of a compound data set. The herein called database fingerprint (DFP) is schematically illustrated in Fig. 1b and further explained throughout this Preliminary Communication. The DFP is inspired on the concept of Shannon entropy (SE) [3] and is based on redundancies present in binary representations. It is well known that the redundancies present in a given signal are the responsible of the information content and therefore of the indirect relation with noise and SE. DFP take advantage of these facts to extract the general pattern of molecular information contained in chemical compound sets represented with any binary fingerprint. As case of study, a DFP was generated for ten data sets of general interest in chemistry with particular emphasis on drug discovery. The basic concept of DFP is illustrated first with a small fingerprint (MACCS keys 166-bits) for relative small data sets (up to 1500 molecules). Then, the application of DFP is shown for a newer and more complex molecular representation (PubChem fingerprints) for larger databases up to 25,000 molecules. Related molecular representation methods like bit fingerprints and different informational content metrics can be complementary to DFP in studies of consensus chemical space characterization [4–7]. One of such approaches is the modal fingerprint. This fingerprint is based on common molecular paths found in chemical sets to determine a unique representation of 2048 bits long that depends in a preset percentage of the database used. This representation can contain, for example, carbonyl or amide functional groups, but also molecular fragments or complete molecular structures [8].

## Methods

### DFP concept and construction

**Fig. 2:**
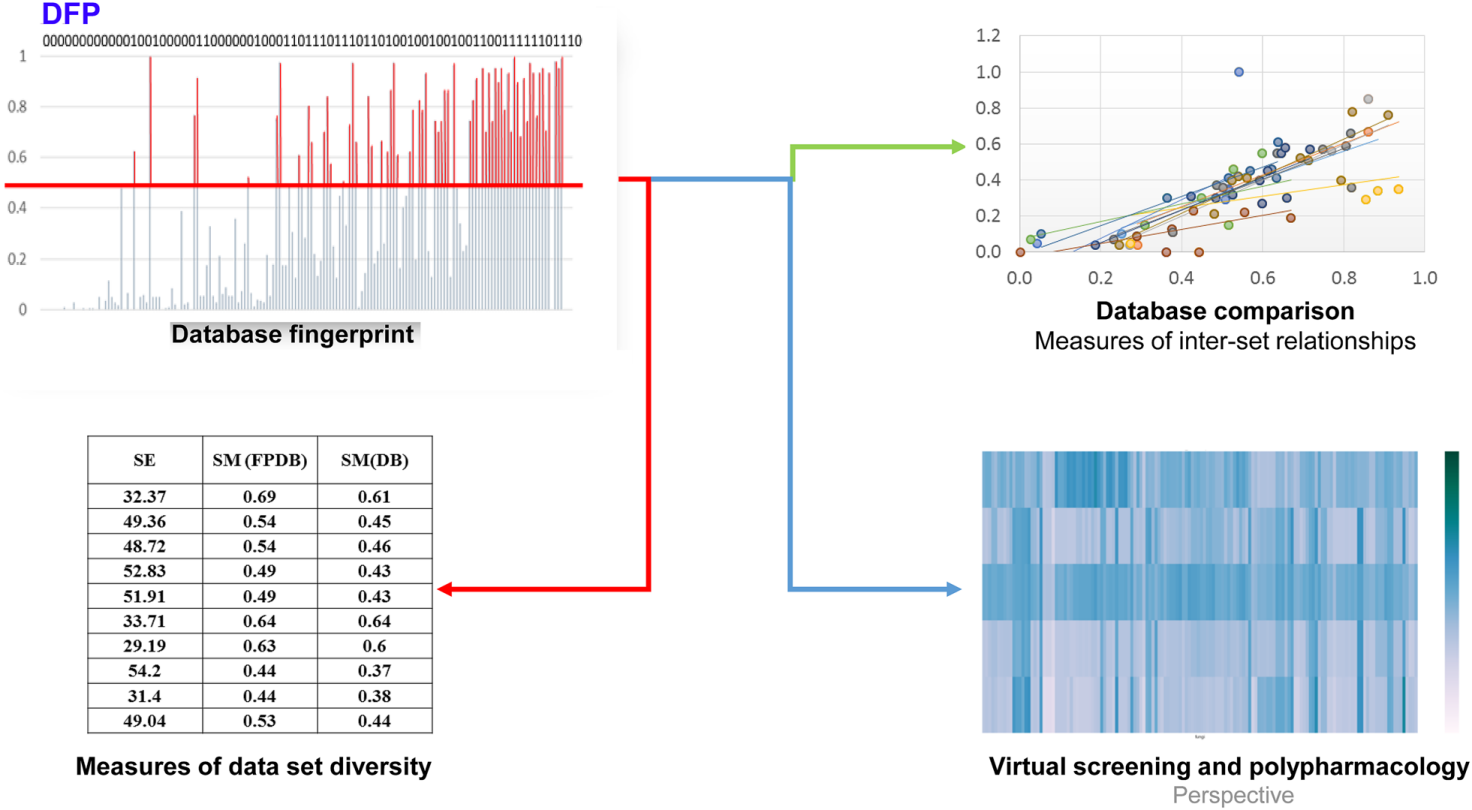
Overview of the approach implemented in this work

The main steps to construct the DFP are shown in Fig. 2. To illustrate the concept of DFP, MACCS keys (166-bits) [2] were calculated for the ten compound data sets in Table 1 using MayaChemTools [9]. As a reference, 1500 binary vectors 166-bit long were generated randomly with the server www.random.org (that uses atmospheric noise to generate random numbers). Since the focus of this work was the generation of a novel fingerprint representation that includes the main features (bit positions) of the compounds in a molecular library, the following approach, inspired on the concept and applications of SE [10, 11] was followed: Firstly, for each binary digit position of the features encoded in the MACCS keys the frequencies and probabilities were recorded. Then, the total SE of the distribution of the 166-bits in the MACCS keys was computed (as a metric of molecular diversity).

**Table 1:** Compound databases used to illustrate the concept of DFP

Database	Type	Size	Mean MACCS keys/Tanimoto	SE^a^
Benzimidazole	In-house	92	0.61	32.37
Epigenetic focused	Commercial	113	0.45	49.36
DNMT1	In-house	566	0.46	48.72
Clinical	Therapeutic target database	837	0.43	52.83
General screening	Commercial (website)	1100	0.43	51.91
Natural products	Natural products	1498	0.64	33.71
Semi-synthetic	Related to natural products	1498	0.60	29.19
Drugs	Approved for clinical use	1490	0.37	54.20
GRAS	Approved in the food industry	1500	0.38	31.40
GDB13	Generated Data Base 13	1500	0.44	49.04

^a^SE: Shannon entropy

To generate the DFP a threshold for the bit probability was established. If the probability for a given bit was greater than the threshold, the bit position was assigned with a number 1. If the probability was equal or lower than the threshold, the bit position was assigned with a number zero. Lastly, to construct the DFP with MACCS keys, two different probability thresholds were explored as first approach: (a) the mean value of the probability distribution of the herein calculated random vectors (0.55) and (b) the mean probability of a data set plus one standard deviation.

To illustrate the concept of the DFP ten data sets were chosen as test cases (Table 1). The compound collections cover a broad range of sizes (ranging from 92 to 1500 molecules) and structural features. Data sets included a small group of 92 synthetic compounds sharing the benzimidazole scaffold (this data set has been used in activity landscape studies [12], a commercial set of 113 molecules for epigenetic drug discovery (‘Epigenetic focused’), an in-house data set with 566 compounds tested as inhibitors of DNA methyltransferase 1 (DNMT1). This set has been used in chemoinformatic analysis of the epigenetic relevant chemical space [13, 14]. Other compound collections used here were 837 molecules in clinical trials (‘Clinical’), a general screening collection (typically used in high-throughput screening—HTS) with 1100 molecules, 1498 natural products and 1498 semi-synthetic compounds, 1490 drugs approved for clinical use [15], 1500 generally recognized as safe (GRAS) compounds [16] and a set of 1500 molecules selected from Generated Data Base 13 (GDB13) available at http://gdb.unibe.ch/downloads/ [17].

### DFP application with PubChem fingerprint and larger data sets

The application of the DFP was applied on 100–25,000 compound databases (Table 2). To this end, we used the PubChem fingerprint that is a newer and more complex molecular representation. For this section we increased the number of compounds for several libraries and included a data set used in HTS with 15,000 molecules (PrimScreen 15 available at http://www.otavachemicals.com/download-compound-libraries/cat_view/110-diversity-sets). The PubChem fingerprint encodes molecular fragments information with 881 binary digits. The list of the substructure encoded on each bit can be accessed at ftp://ncbi.nlm.nih.gov/pubchem/specifications/pubchem_fingerprints.txt. This molecular representation was selected to calculate the bit position frequencies and probability distributions to construct the DFP for the original databases.

**Table 2:** Compound databases used to show the application of DFP

Database	Type	Size
Benzimidazole	In-house	92
Epigenetic focused	Commercial	113
DNMT1	In-house	566
Clinical	Therapeutic target database	830
General screening	Commercial (website)	1100
Natural products	Natural products	4460
Semi-synthetic	Related to natural products	25,327
Drugs	Approved for clinical use	1462
GRAS	Approved in the food industry	2244
PrimScreen15	PrimaryScreen 15	14,489
FDA	Approved for clinical use	1621

For this part, three different thresholds (0.5, 0.6 and 0.7), the informational significant bit positions were selected using Differential Shannon Entropy [18] implemented in the IMMAN package software [19]. The probability distribution and relation between classical Shannon entropy average, DFP/Tanimoto similarity and k-mean clustering of the informational significant bit positions was studied.

## Results and discussion

This section is organized in two major parts. First, the concept of DFP is discussed using MACCS keys for compound data sets up to 1500 compounds. The second part shows an application of DFP with PubChem fingerprints for larger data sets.

### Distribution of binary fingerprint: SE as metric of database diversity

**Fig. 3:**
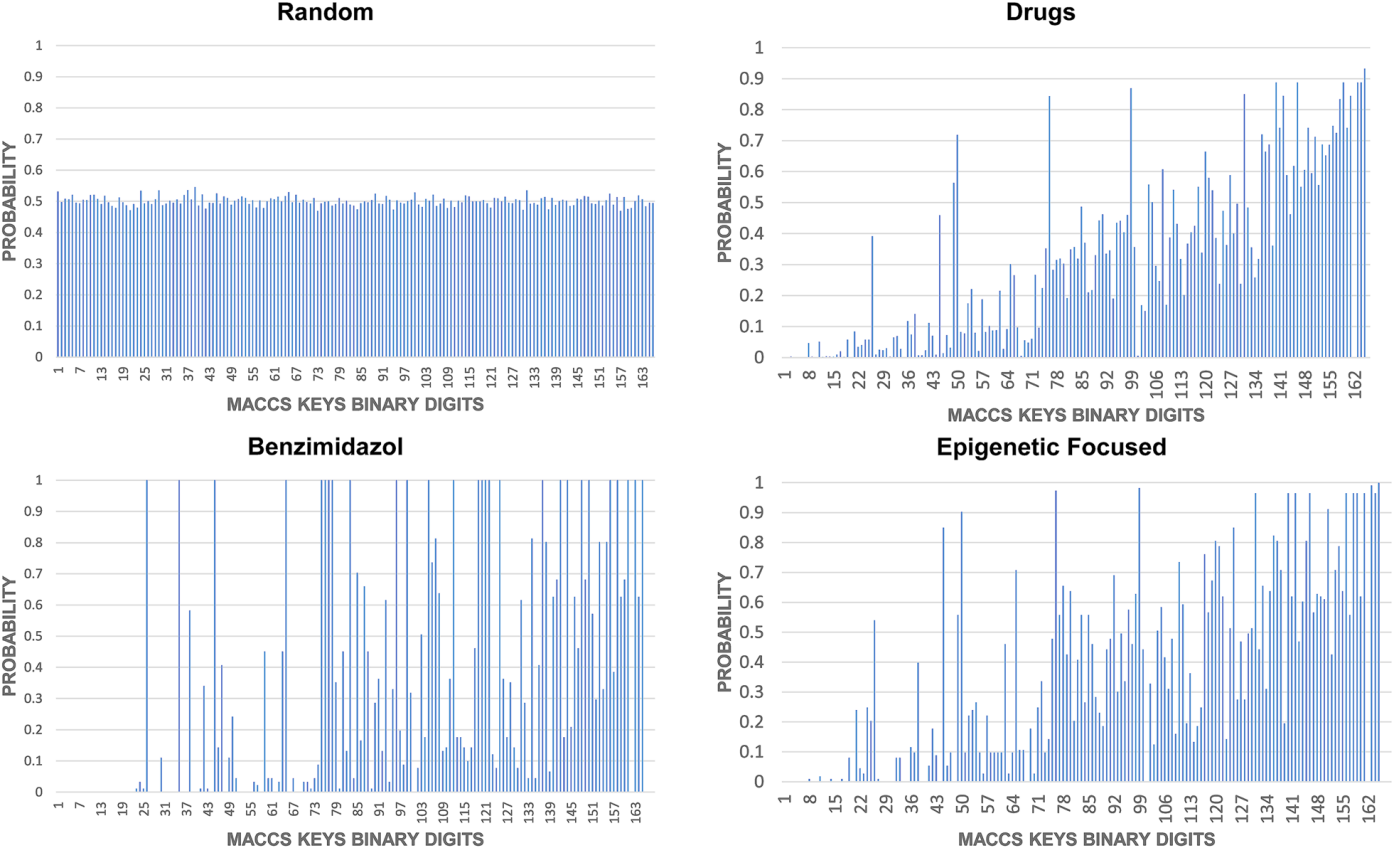
Probability distributions of MACCS keys (166-bits) of representative data sets studied in this work. The number of compounds, mean MACCS keys/Tanimto similarity, and Shannon entropy (SE) are shown

Figure 3 shows the probability distributions of MACCS keys (166-bits) for three representative data sets (drugs, benzimidazoles, and Epigenetic-focused) plus the randomly generated binary fingerprints as a reference. The probability distributions of the other compound data sets are shown in Additional file 1: Fig. S1. The corresponding SE values for each probability distribution is shown in each group and are further reported in Table 1 for all data sets. In addition, Table 1 summarizes the mean similarity value using the MACCS keys fingerprints and Tanimoto index (MACCS keys/Tanimoto similarity) of all ten data sets. Table 1 and Fig. 3; Additional file 1: Fig. S1 show that each data set had different values of SE that was associated with the mean MACCS keys/Tanimoto similarity.

**Fig. 4:**
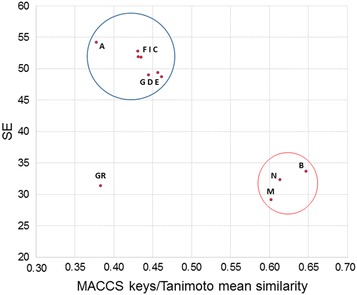
Relationship Shannon Entropy and MACCS keys/Tanimoto similarity for the ten compound data sets in Table 1. *A* drugs, *I* general screening, *C* clinical, *G* GDB13, *D* DNMT1, *E* epigenetic focused, *M* semi-synthetic, *N* natural products, *B* benzimidazole, *GR* GRAS, *R* random

Figure 4 shows the relationship between SE and mean MACCS keys/Tanimoto similarity. The plot shows that high SE is associated with high intra-set diversity i.e., low similarity. Likewise, lower SE is associated with high similarity. Of note, SE is not a magnitude that can be expressed in terms of an absolute scale because no upper limit boundaries are known. A general observation is that high SE is an indicative that it is less likely that two compounds in the data set have similar fingerprint representation. If this observation is repeated for many pairs of compounds in the data set, then the overall similarity of the compound data set is low and the mean similarity of the data set is expected to be low. In contrast, if the overall SE of the data set is (relatively) low, it is likely that two molecules in the data set have similar fingerprint representation. Therefore, it is expected that the overall diversity of the data set is (relatively) low e.g., the overall similarity of the compound data set is high. This general trend was observed for nine out of ten data sets.

A notable exception was the GRAS set: SE of the MACCS keys has a relative low value (30) but the data set has high diversity (as measured with MACCS keys/Tanimoto <0.40). In other words, despite the fact that there is a relative low entropy in the fingerprint representation of GRAS, it happens that the likelihood that two compounds share similar fingerprint representation is low. It is worth noting that MACCS keys/Tanimoto captures pair-wise relationships that are not directly captured by the SE of the entire fingerprint. A second notable exception was the random set that had, as expected, the highest SE value (above 80) but MACCS keys/Tanimoto similarity of 0.33. The distinct feature of GRAS (as compared to the other data sets considered in this work) can be related to the particular structural features of molecules in this data set. It has been shown that GRAS molecules have a high content of aliphatic chain and has a low diversity of molecular scaffolds [20]. It should also be considered that MACCS keys is unable to capture the particular features of GRAS compounds.

The plot in Fig. 4 shows two main clusters that group together the different data sets. These databases can be related through the nature of the compounds in each cluster. In the larger cluster (upper left), all the data sets, with exception of GDB13, are related to synthetic bioactive molecules. While the small cluster contains data sets that include natural products, semi-synthetic natural products and benzimidazole derivatives, all present in living organisms.

Based on the above results, it can be suggested that SE of the probability distributions of MACCS keys (166-bits) can be used as an additional criterion to rapidly assess the fingerprint-based diversity of compound data sets. Of course, additional metrics and criteria e.g., scaffold diversity, should be considered for a comprehensive assessment of the structural diversity of data sets [21]. It is worth noting that the concept of SE was initially used to measure the content of information in particular messages [3]. Nowadays, along with similarity and molecular scaffolds, SE has been implemented to measure scaffold diversity [10, 22]. In chemoinformatics, SE is also related to the generation of many kinds of molecular representations based on graph theory and virtual similarity searches, among others [23, 24]. In particular, SE has been used previously to determine the similarity between a given molecule and a focused library [24]. In that approach, Wang et al. calculated the variation of SE of a focused library with and without a given compound to determine their similarity with the redundant futures present in the database.

### DFP

As described above, 166-bit long DFP were generated for all ten compound data sets in Table 1. Representative DFP of selected data sets are shown in Additional file 1: Table S1. Two different thresholds were used to determine the limit redundancy value, the mean probability of a random distribution and the inter-mean plus one database standard deviation (vide supra). As described below, to select the most representative threshold value, a comparison with city block distance was performed. Using this criteria one DFP per database was calculated with the different thresholds, resulting in the selection of the mean probability of a random distribution as a final threshold.

### DFP and inter-set relationships

Table 3 shows the city block distance [1] between the data sets considering the newly developed DFP. A 2D visualization of the distance matrix is presented in the Additional file 1: Fig. S2.

**Table 3:** Inter-set relationships of the compound data sets computed with the database fingerprint (DFP) and city block distance

	Random	GDB13	DNMT1	GRAS	NP	SS	Benz	GS	Drugs	Clinical	EF
Random	0										
GDB13	54	0									
DNMT1	51	27	0								
GRAS	67	27	42	0							
NP	63	39	24	48	0						
SS	65	32	34	22	43	0					
Benz	64	35	33	43	32	46	0				
GS	49	23	12	37	24	32	31	0			
Drugs	49	19	17	30	29	27	33	10	0		
Clinical	47	23	13	38	25	32	32	4	9	0	
EF	50	26	12	42	24	37	32	8	15	9	0

*NP* natural products, *SS* semi-synthetic, *Benz* benzimidazole, *GS* general screening, *EF* epigenetic focused

As expected, the randomly generated set was the most distant i.e., most dissimilar, to the other ten data sets with real molecules. In agreement with previous publications [13, 14] there was a small distance between compounds in the clinic (‘Clinical’) and general screening and approved drugs. Similarly, there was a small distance between the commercial molecules focused on epigenetic targets (‘Epigenetic focused’) and compounds for general screening and molecules in the ‘Clinic’. Indeed, it can be expected a large overlap between the chemical spaces of all these data sets using MACCS keys from which the DFP was designed. In contrast, after random, GRAS compounds were the second most distant to all other data sets considered in this study. This is consistent with previous results that support that GRAS molecules are dissimilar to other databases commonly used in drug discovery using MACCS keys [25].

Taken together the results in Table 3, further visualized in Additional file 1: Fig. S2, can be concluded that the newly DFP is a reasonable approximation of the fingerprint-based representation of a molecular database. Similar trends between the inter-set relationships were obtained with the DFP and the Tanimoto coefficient (Additional file 1: Table S2 and Fig. S3), and the inter-set relationships calculated with MACCS keys and the Tanimoto coefficient (Additional file 1: Fig. S4).

### DFP and intra-set relationship

Table 4 shows the relationship between the intra-set mean similarities computed with two strategies, namely; a classical approach calculated the pair-wise mean similarity with MACCS keys/Tanimoto coefficient. The second approach was an approximation of the intra-set similarity using the newly proposed DFP: for each data set, the similarity based on the DFP was calculated as the mean similarity between the MACCS keys representation of each compound and the DFP of the data set. Results summarized in Table 4 (and plotted in Additional file 1: Fig. S5) show a direct relationship between these two values supporting the hypothesis that DFP was able to retain the general information contained in a given compound data set. Even if DFP underestimated the similarity values (Table 4), it was a reasonable tool to estimate the intra-set molecular diversity, since these comparison studies are relative to the databases.

**Table 4:** Intra-set mean similarity of the compound data sets

Date set	Mean similarity (MACCS keys)^a^	Mean similarity (DFP)^b^
Benzimidazole	0.61	0.69
Epigenetic focused	0.45	0.54
DNMT1	0.46	0.54
Clinical	0.43	0.49
General screening	0.43	0.49
Natural products	0.64	0.64
Semi-synthetic	0.60	0.63
Drugs	0.37	0.44
GRAS	0.38	0.44
GDB13	0.44	0.53

^a^Pair-wise mean similarity calculated with MACCS keys/Tanimoto coefficient

^b^Calculated as the mean similarity between the MACCS keys representation of each compound and the DFP of the data set

### DFP application with PubChem fingerprint and larger data sets

**Fig. 5:**
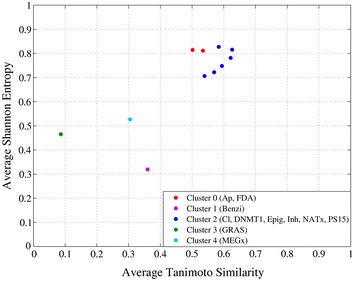
Relationship Shannon entropy and DFP/Tanimoto similarity and k-mean Euclidean clustering for the ten compound data sets in Table 2 at threshold of 0.5 threshold value

For three different thresholds (0.5, 0.6 and 0.7) the informational significant bit positions of PubChem, 198, 180, and 159 respectively, were selected using Differential Shannon Entropy implemented in IMMAN package software. Figure 5 shows the classical Shannon entropy average versus the average DFP/Tanimoto Similarity based in the 198 information significant bit positions obtained with a 0.5 threshold with IMMAN software. Figure 5 also displays the databases cluster membership on five clusters obtained with k-mean Euclidean distances implemented in WEKA software [26].

Similar to Figs. 4, 5 shows two main clusters that group together different data sets that contain chemically related compounds. For instance, in the larger cluster colored blue, all the data sets, with exception of PS15, are related to synthetic bioactive molecules. While the small two-member clusters, in red color, group FDA and Approved datasets. The one-member clusters correlates with the previously reported distinct nature of GRAS, MEGx, and Benzi compounds.

**Fig. 6:**
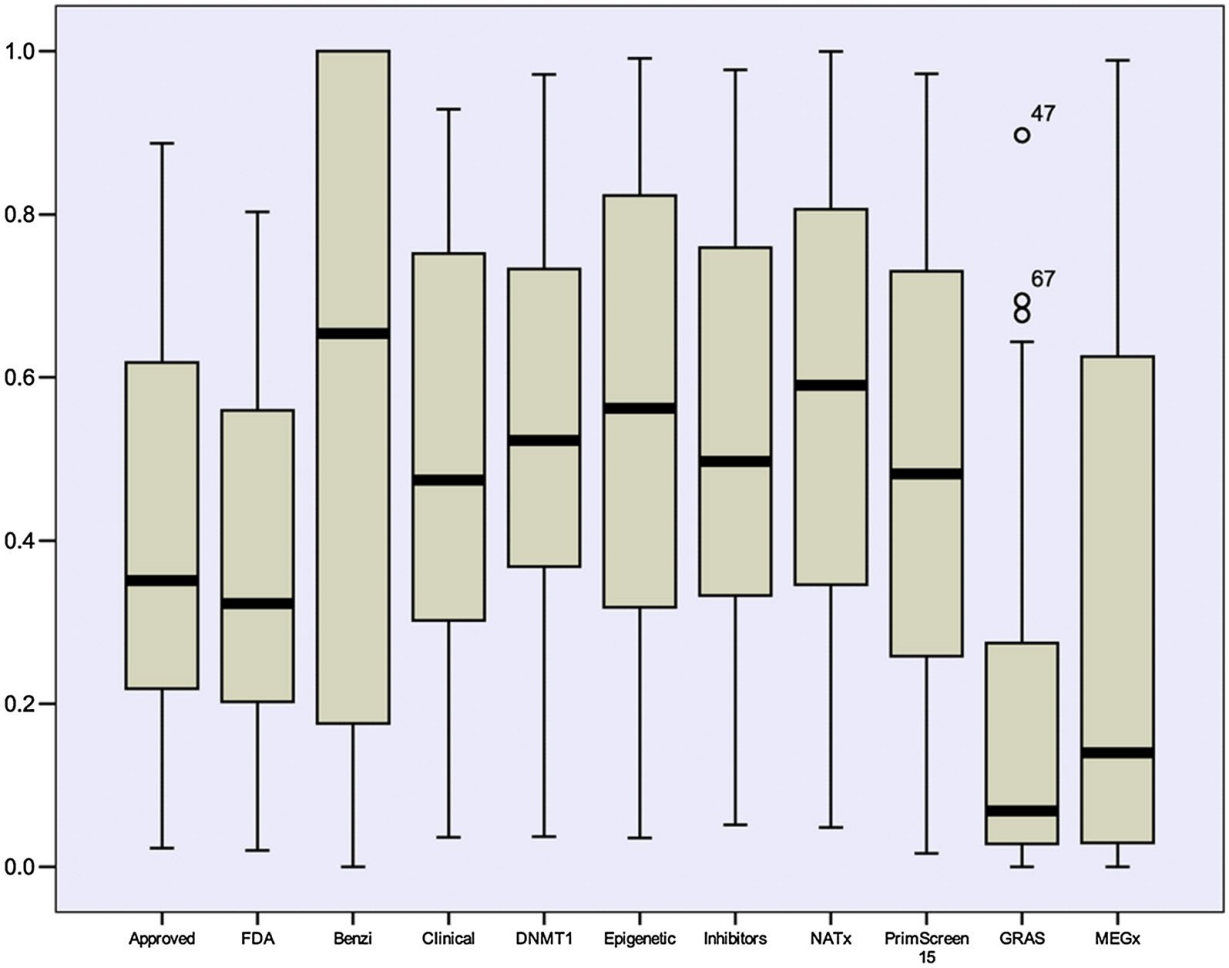
Probability distribution of the 198 significant bit positions recovered from the original databases represented by PubChem fingerprint at threshold of 0.5

This general grouping of compound data sets in Fig. 5 is consistent with the probability distribution of the 198 significant bit positions recovered from the original databases represented by PubChem fingerprints. In Fig. 6 the datasets probability distributions can by grouped in a similar way to the cluster membership illustrated in Fig. 5.

The same analysis was applied for 0.6 and 0.7 DFP thresholds. The implementation of this cutoff criteria led to a significant decrease in the resolution of the DFP to distinguish differences between the databases studied. The respective probability distributions and classical Shannon entropy average versus the average DFP/Tanimoto Similarity plots, with k-mean clustering, can be found in the Additional file 1: Figs. S6–S9.

## Conclusions and perspectives

In this Preliminary Communication we introduced the DFP as an approach to generate a binary fingerprint representation of a compound collection with a fixed size. The new fingerprint has the ability to include the main structural futures of the molecules in the data set. The construction of the DFP is based on the distribution of the probabilities of each position in a given binary fingerprint of fixed length. A test cases, DFP were generated for ten compound data sets of different size using, as an example, a short and commonly used fingerprint representation: MACCS keys (166-bits). The application of DFP is also illustrated for large molecular libraries using PubChem fingerprints, with a total of 881-bits. DFP for compound data sets with a broad range size (ranging from 100 to 25,000 molecules) were calculated using three different threshold values to explore the fingerprint behavior with respect to database size, diversity, cutoff criteria and different content of information metrics. It was concluded that DFPs are reasonable representations of the compound data sets to measure the intra- and inter-set relationships. One of the principal perspectives of DFP is its performance in virtual screening and library design applications. Despite the fact that a quantitative analysis of the advantages of DFP over other fingerprints in terms of computer time is beyond the scope of this work [the comparison will largely depend on the specific fingerprints compared, compound databases and computer(s) processors] is clear that DFP saves time because they are pre-calculated and stored for later applications.

## Additional file

Additional file 1:Table S1. DFPs of representative data sets used in this work. **Table S2**. Inter-set relationship computed with the newly developed database fingerprint using DFP/Tanimoto coefficient. **Fig. S1** Distributions of MACCS keys (166-bits) of selected data sets studied in this work (others are shown in the main text). **Fig. S2** Visual representation of the distance matrix comparing inter-set relationships of the compound data sets computed with the database fingerprint (DFP) and city block distance. **Fig. S3** Relationship between inverse normalized city block distance and Tanimoto similarity using the DFP. **Fig. S4** Inter-set relationships of the compound data sets computed with MACCS keys and the Tanimoto coefficient. **Fig. S5** Relationship between mean similarities computed with MACCS keys and DFP. **Fig. S6** Relationship Shannon Entropy and DFP/Tanimoto similarity and k-mean Euclidean clustering for the ten compound data sets in Table 2 at threshold of 0.6. **Fig. S7** Probability distribution of the 198 significant bit positions recovered from the original databases represented by PubChem fingerprint at threshold of 0.6.**Fig. S8** Relationship Shannon Entropy and DFP/Tanimoto similarity and k-mean Euclidean clustering for the ten compound data sets in Table 2 at threshold of 0.7. **Fig. S9** Probability distribution of the 198 significant bit positions recovered from the original databases represented by PubChem fingerprint at threshold of 0.7.

## Abbreviations

DFP: database fingerprint
DNMT1: DNA methyltransferase 1
GDB13: Generated Data Base 13
GRAS: generally recognized as safe
HTS: high-throughput screening
MACCS: molecular access system
SE: Shannon entropy
FDA: FDA approved for clinical use
PS15: PrimaryScreen 15
DFP/Tanimoto similarity: similarity value using the DFP representation and Tanimoto index
MACCS keys/Tanimoto similarity: similarity value using the MACCS keys fingerprints and Tanimoto index
