# Supplementary material for: Database fingerprint (DFP): an approach to represent molecular databases
Source: J Cheminform. 2017 Feb 6;9:9. doi: 10.1186/s13321-017-0195-1 (PMC5293704; doi:10.1186/s13321-017-0195-1)
Supplement: Supplementary file 1 — Additional file 1:Table S1. DFPs of representative data sets used in this work. Table S2. Inter-set relationship computed with the newly developed database fingerprint using DFP/Tanimoto coefficient. Fig. S1 Distributions of MACCS keys (166-bits) of selected data sets studied in this work (others are shown in the main text). Fig. S2 Visual representation of the distance matrix comparing inter-set relationships of the compound data sets computed with the database fingerprint (DFP) and city block distance. Fig. S3 Relationship between inverse normalized city block distance and Tanimoto similarity using the DFP. Fig. S4 Inter-set relationships of the compound data sets computed with MACCS keys and the Tanimoto coefficient. Fig. S5 Relationship between mean similarities computed with MACCS keys and DFP. Fig. S6 Relationship Shannon Entropy and DFP/Tanimoto similarity and k-mean Euclidean clustering for the ten compound data sets in Table 2 at threshold of 0.6. Fig. S7 Probability distribution of the 198 significant bit positions recovered from the original databases represented by PubChem fingerprint at threshold of 0.6.Fig. S8 Relationship Shannon Entropy and DFP/Tanimoto similarity and k-mean Euclidean clustering for the ten compound data sets in Table 2 at threshold of 0.7. Fig. S9 Probability distribution of the 198 significant bit positions recovered from the original databases represented by PubChem fingerprint at threshold of 0.7. [file 13321_2017_195_MOESM1_ESM.pdf]

SUPPORTING INFORMATION

Database Fingerprint (DFP): An approach to represent molecular databases

Eli Fernández-de Gortari[*,1], César R. García-Jacas[2], Karina Martinez-Mayorga[2], José L. Medina-Franco[*,1]

Contents

		Page
**Table S1**	DFPs of representative data sets used in this work.	S2
**Table S2**	Inter-set relationship computed with the newly developed database fingerprint using DFP/Tanimoto coefficient.	S4
**Fig. S1**	Distributions of MACCS keys (166-bits) of selected data sets studied in this work (others are shown in the main text).	S5
**Fig. S2**	Visual representation of the distance matrix comparing inter-set relationships of the compound data sets computed with the database fingerprint (DFP) and city block distance.	S6
**Fig. S3**	Relationship between inverse normalized city block distance and Tanimoto similarity using the DFP.	S7
**Fig. S4**	Inter-set relationships of the compound data sets computed with MACCS keys and the Tanimoto coefficient.	S8
**Fig. S5**	Relationship between mean similarities computed with MACCS keys and DFP.	S9
**Fig. S6**	Relationship Shannon Entropy and DFP/Tanimoto similarity and k-mean Euclidean clustering for the ten compound data sets in Table 2 at threshold of 0.6.	S10
**Fig. S7**	Probability distribution of the 198 significant bit positions recovered from the original databases represented by PubChem fingerprint at threshold of 0.6.	S11
**Fig. S8**	Relationship Shannon Entropy and DFP/Tanimoto similarity and k-mean Euclidean clustering for the ten compound data sets in Table 2 at threshold of 0.7.	S12
**Fig. S9**	Probability distribution of the 198 significant bit positions recovered from the original databases represented by PubChem fingerprint at threshold of 0.7.	S13

Table S1. DFPs of representative data sets used in this work.

Benimidazoles

0	0	0	0	0	0	0	0	0	0
0	0	0	0	0	0	0	0	0	0
0	0	0	0	0	1	0	0	0	0
0	0	0	0	1	0	0	1	0	0
0	0	0	0	1	0	0	0	0	0
0	0	0	0	0	0	0	0	0	0
0	0	0	0	1	0	0	0	0	0
0	0	0	0	1	1	1	1	0	0
0	0	1	0	1	0	1	0	0	0
0	0	1	0	0	1	0	0	1	0
0	0	0	0	1	1	1	1	0	0
0	1	0	0	0	0	0	0	1	1
1	1	0	0	1	0	0	0	0	0
1	0	0	1	0	0	1	1	0	1
1	1	0	1	0	1	0	1	1	1
1	0	1	0	1	1	0	1	1	1
1	0	1	1	1	0				

Epigenetic focused

0	0	0	0	0	0	0	0	0	0
0	0	0	0	0	0	0	0	0	0
0	0	0	0	0	0	0	0	0	0
0	0	0	0	0	0	0	0	0	0
0	0	0	0	1	0	0	0	1	1
0	0	0	0	0	0	0	0	0	0
0	0	0	0	1	0	0	0	0	0
0	0	0	0	0	1	1	1	0	1
0	0	1	0	1	0	0	0	0	0
0	1	0	0	0	1	0	1	1	0
0	0	0	0	1	0	0	0	0	1
1	0	0	0	0	0	1	1	1	1
1	1	0	0	1	0	0	0	0	0
1	0	1	0	1	1	1	1	0	1
1	1	0	1	1	1	1	1	1	1
1	0	1	1	1	1	1	1	1	1
1	0	1	1	1	0				

DNMT1

0	0	0	0	0	0	0	0	0	0
0	0	0	0	0	0	0	0	0	0
0	0	0	0	0	1	0	0	0	0
0	0	0	0	0	0	0	0	0	0
0	0	0	0	1	0	0	0	0	1
0	0	0	0	0	0	0	0	0	0
0	0	0	0	1	0	0	0	0	0
0	0	0	0	1	1	0	1	0	1
0	0	1	0	1	0	0	0	0	0
0	1	0	0	1	1	1	0	1	0
0	0	0	0	1	0	0	0	0	1
0	0	0	0	0	0	1	0	1	1
1	1	0	0	1	0	1	0	0	0
1	0	1	0	1	1	1	1	0	1
0	1	1	0	1	1	0	1	0	1
1	1	1	1	1	1	1	1	1	0
1	0	1	1	1	0				

Table S2. Inter-set relationship computed with the newly developed database fingerprint using DFP/Tanimoto coefficient.

	Random	GDB13	DNMT1	GRAS	NP	SS	Benz	GS	Drugs	Clinical	EF
Random	0										
GDB13	0.0	1.0									
DNMT1	0.0	0.4	1.0								
GRAS	0.0	0.4	0.2	1.0							
NP	0.0	0.3	0.7	0.2	1.0						
SS	0.0	0.4	0.4	0.4	0.4	1.0					
Benz	0.0	0.5	0.5	0.2	0.5	0.3	1.0				
GS	0.0	0.5	0.6	0.3	0.6	0.5	0.5	1.0			
Drugs	0.0	0.6	0.5	0.4	0.5	0.5	0.4	0.7	1.0		
Clinical	0.0	0.5	0.6	0.3	0.6	0.5	0.5	0.9	0.8	1.0	
EF	0.0	0.4	0.7	0.3	0.6	0.4	0.6	0.8	0.6	0.8	1.0

NP: Natural products, SS: Semi-synthetic, Benz: Benzimidazole, GS: General screening, EF: Epigenetic focused.

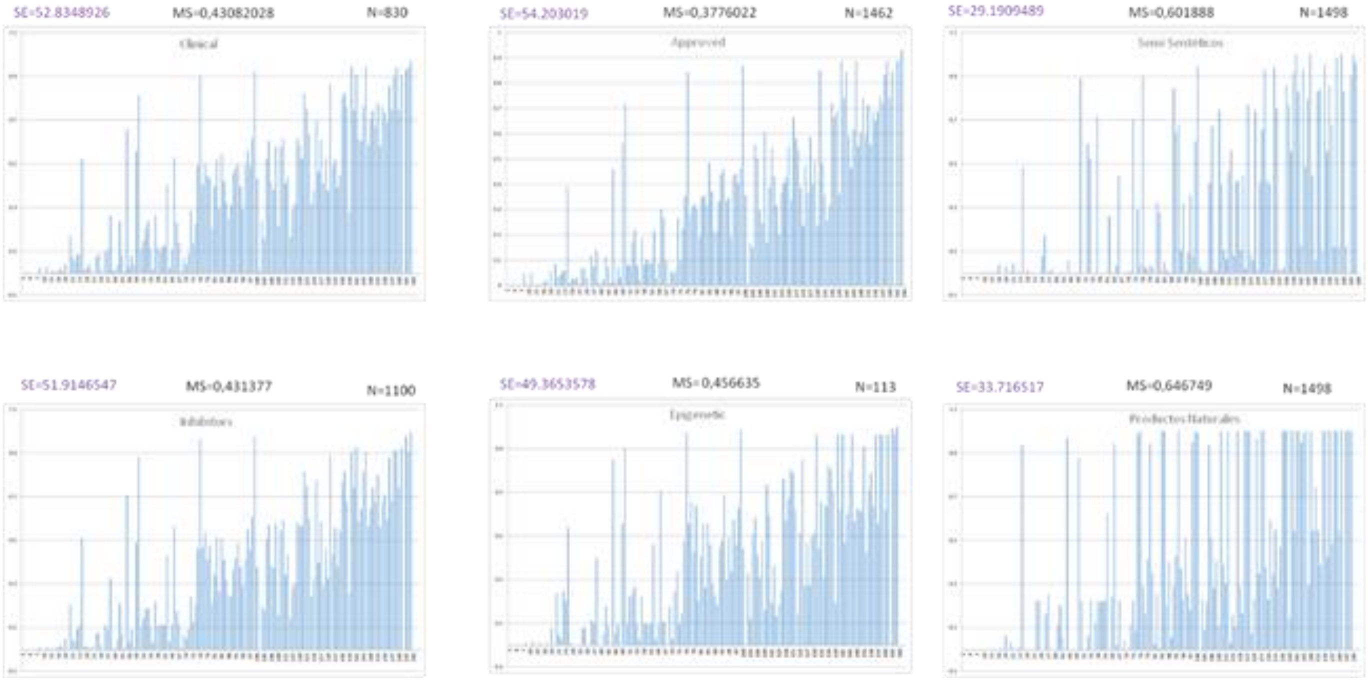

Fig. S1 Distributions of MACCS keys (166-bits) of selected data sets studied in this work (others are shown in the main text).

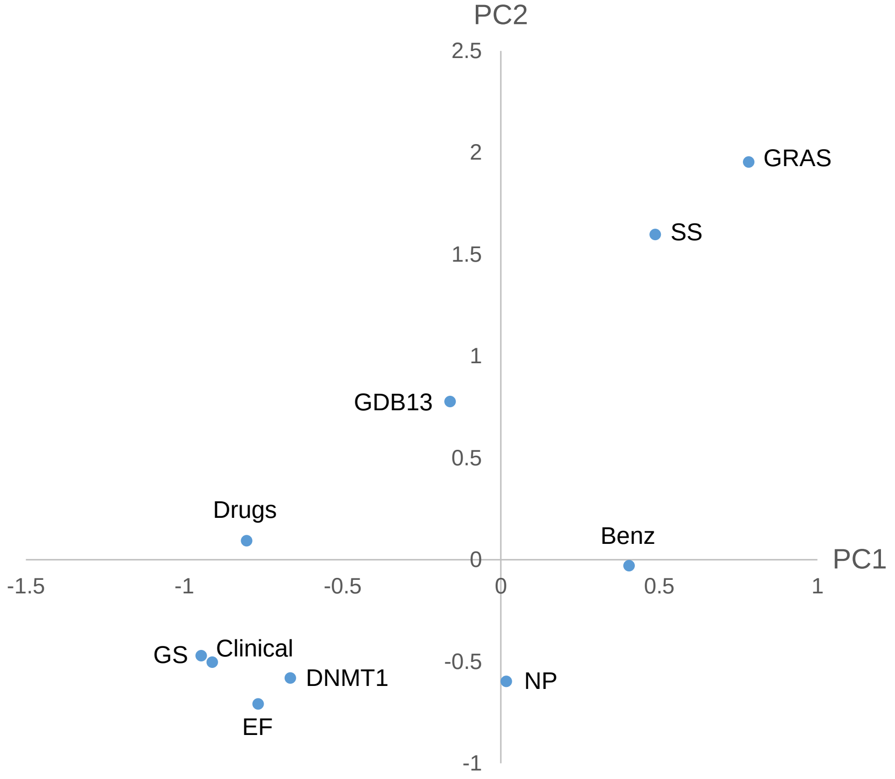

Fig. S2 Visual representation of the distance matrix comparing inter-set relationships of the compound data sets computed with the database fingerprint (DFP) and city block distance. The two-dimensional plot was generated by means of principal component analysis. The variance captured by the first two principal components is 77 %.

A

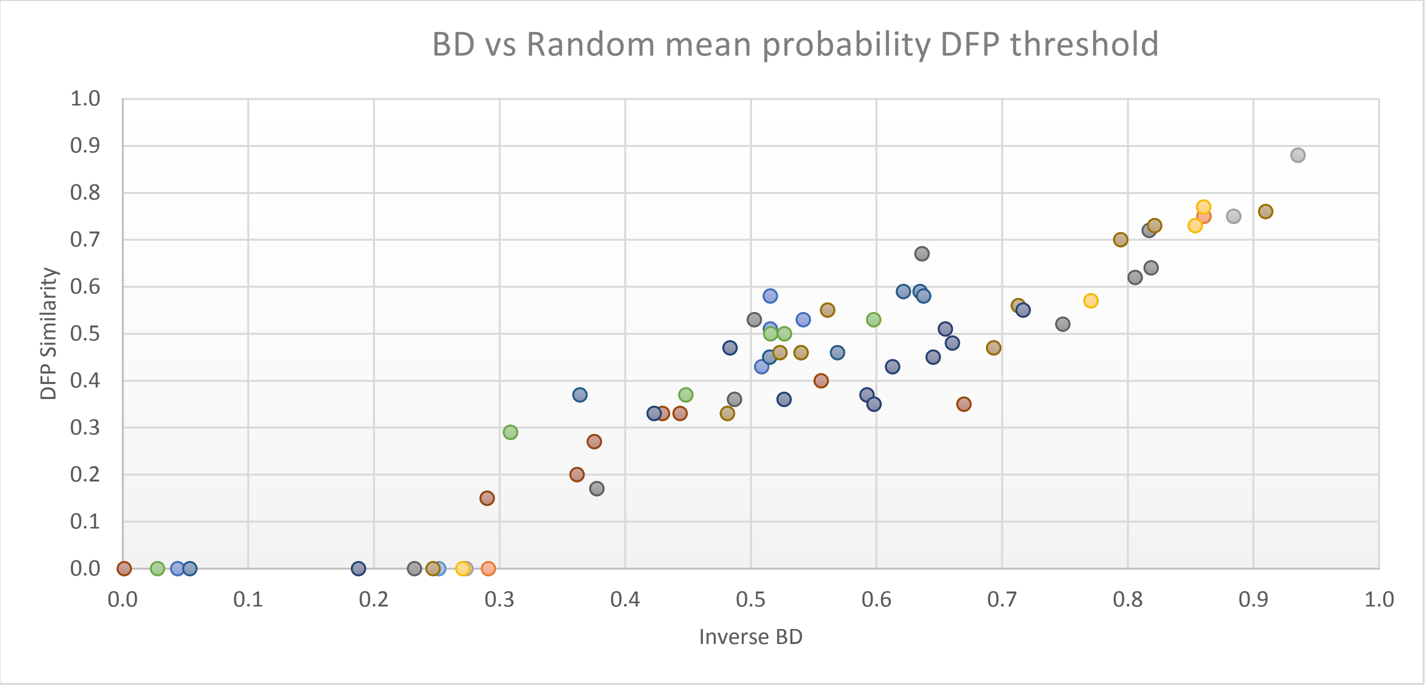

B

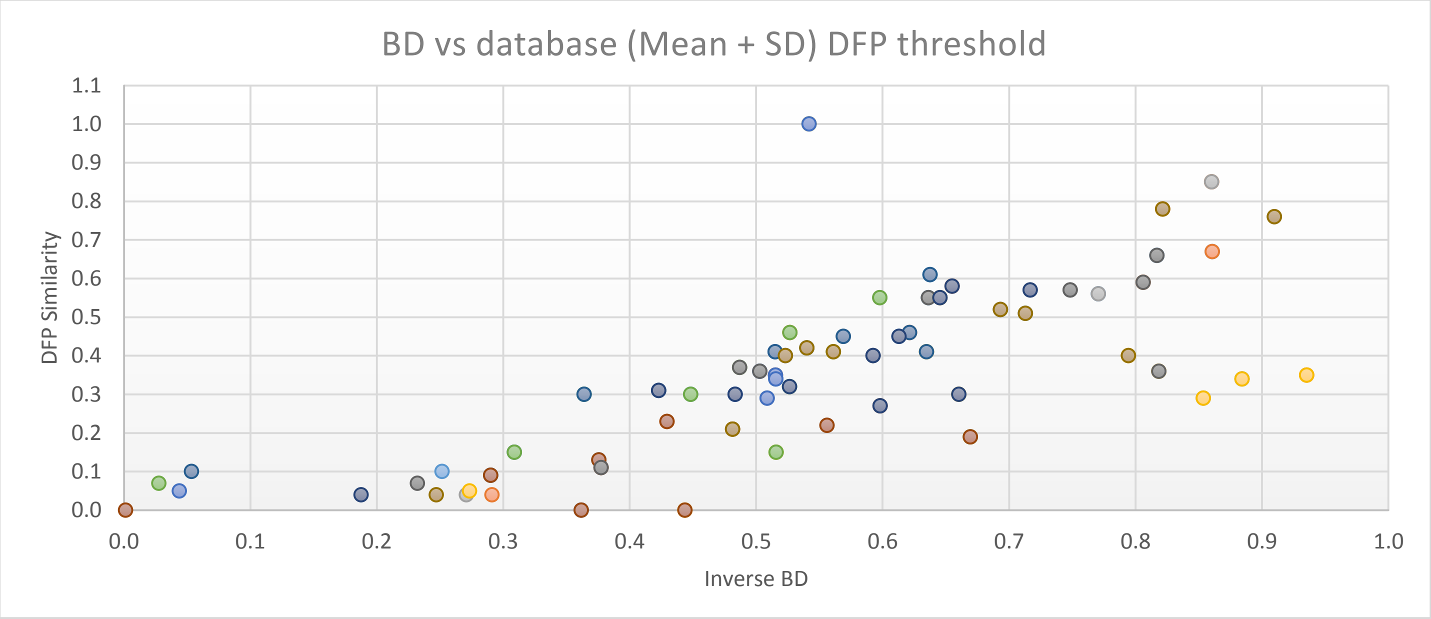

Fig. S3 Relationship between inverse normalized city block distance and Tanimoto similarity using the DFP.

	Random	GDB13	DNMT1	GRAS	NP	SS	Benz	GS	Drugs	Clinical	EF
Random	0										
GDB13	0.0	1.0									
DNMT1	0.0	0.4	1.0								
GRAS	0.0	0.4	0.2	1.0							
NP	0.0	0.3	0.7	0.2	1.0						
SS	0.0	0.4	0.4	0.4	0.4	1.0					
Benz	0.0	0.5	0.5	0.2	0.5	0.3	1.0				
GS	0.0	0.5	0.6	0.3	0.6	0.5	0.5	1.0			
Drugs	0.0	0.6	0.5	0.4	0.5	0.5	0.4	0.7	1.0		
Clinical	0.0	0.5	0.6	0.3	0.6	0.5	0.5	0.9	0.8	1.0	
EF	0.0	0.4	0.7	0.3	0.6	0.4	0.6	0.8	0.6	0.8	1.0

NP: Natural products, SS: Semi-synthetic, Benz: Benzimidazole, GS: General screening, EF: Epigenetic focused.

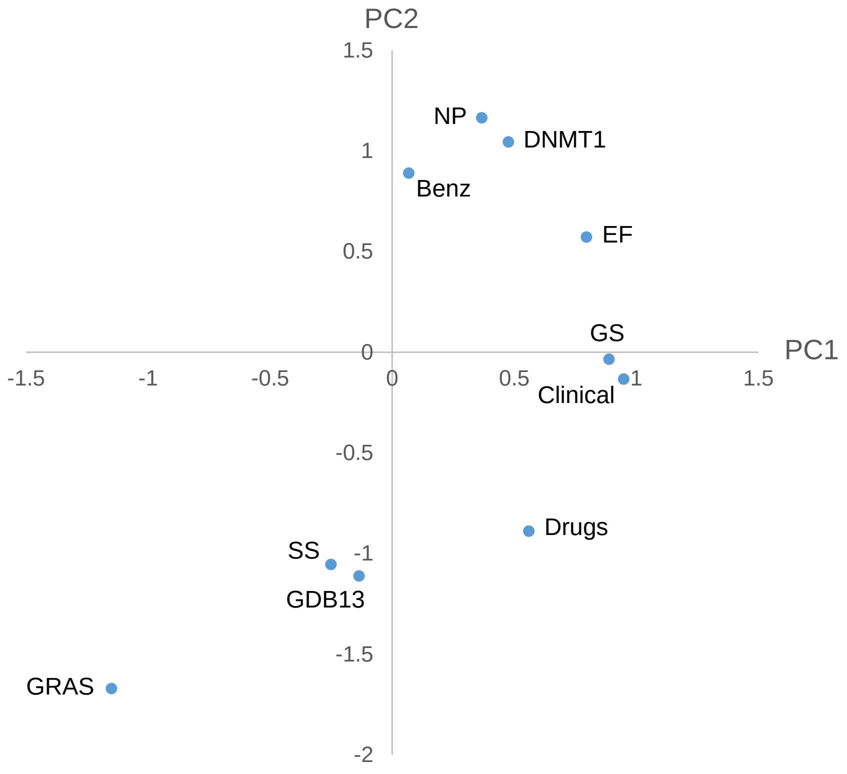

Fig. S4 Inter-set relationships of the compound data sets computed with MACCS keys and the Tanimoto coefficient. Figure shows the similarity matrix and a two-dimensional plot generated by principal component analysis. The variance captured by the first two principal components is 73 %.

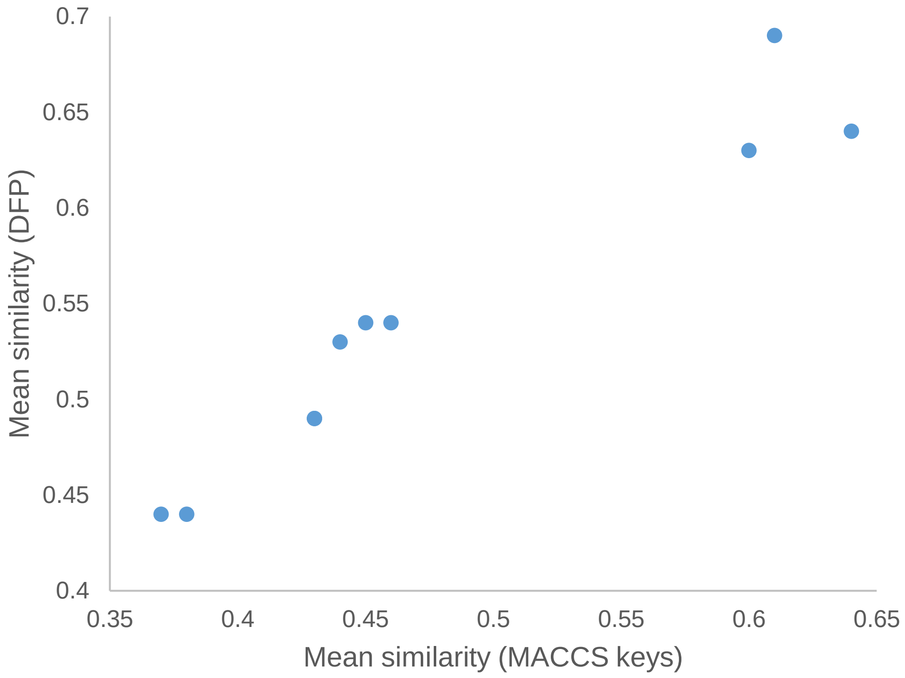

Fig. S5 Relationship between mean similarities computed with MACCS keys and DFP.

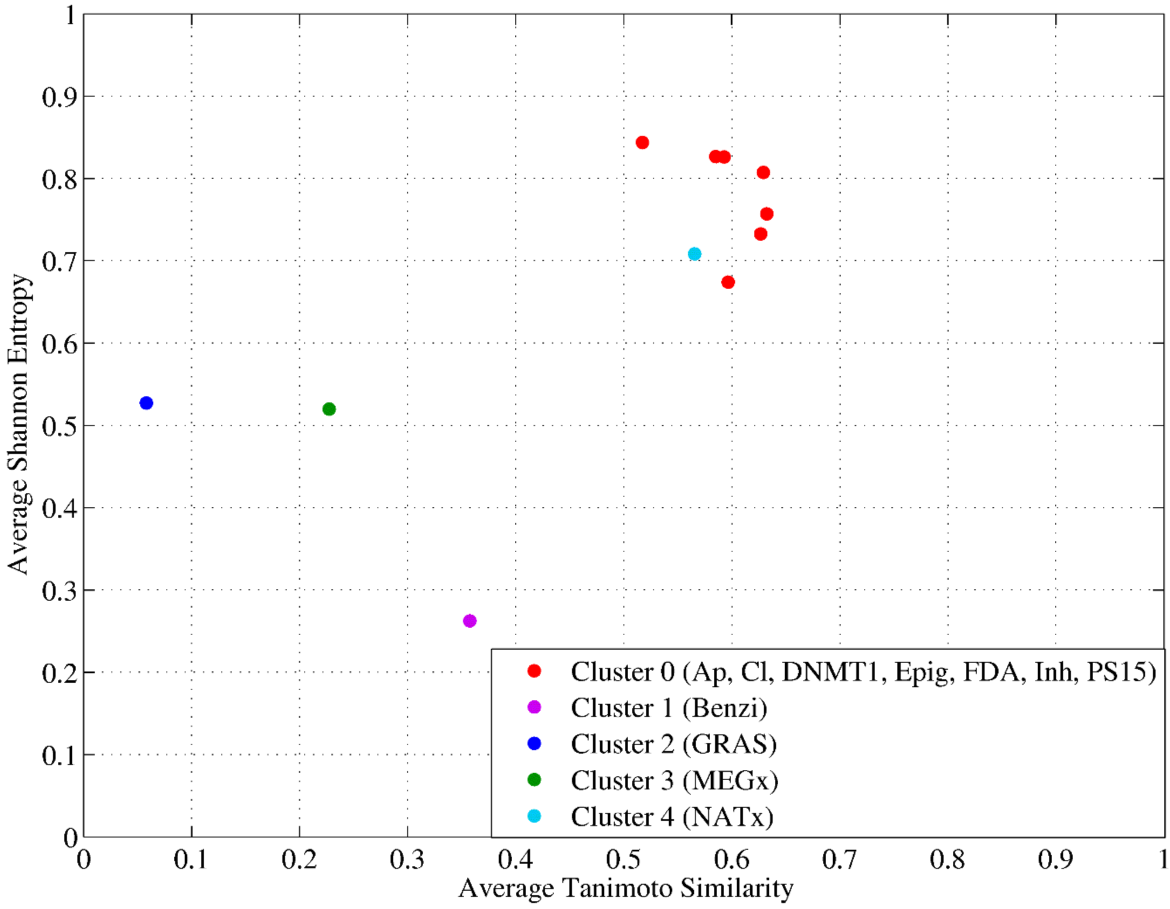

Fig. S6 Relationship Shannon Entropy and DFP/Tanimoto similarity and k-mean Euclidean clustering for the ten compound data sets in Table 2 at threshold of 0.6.

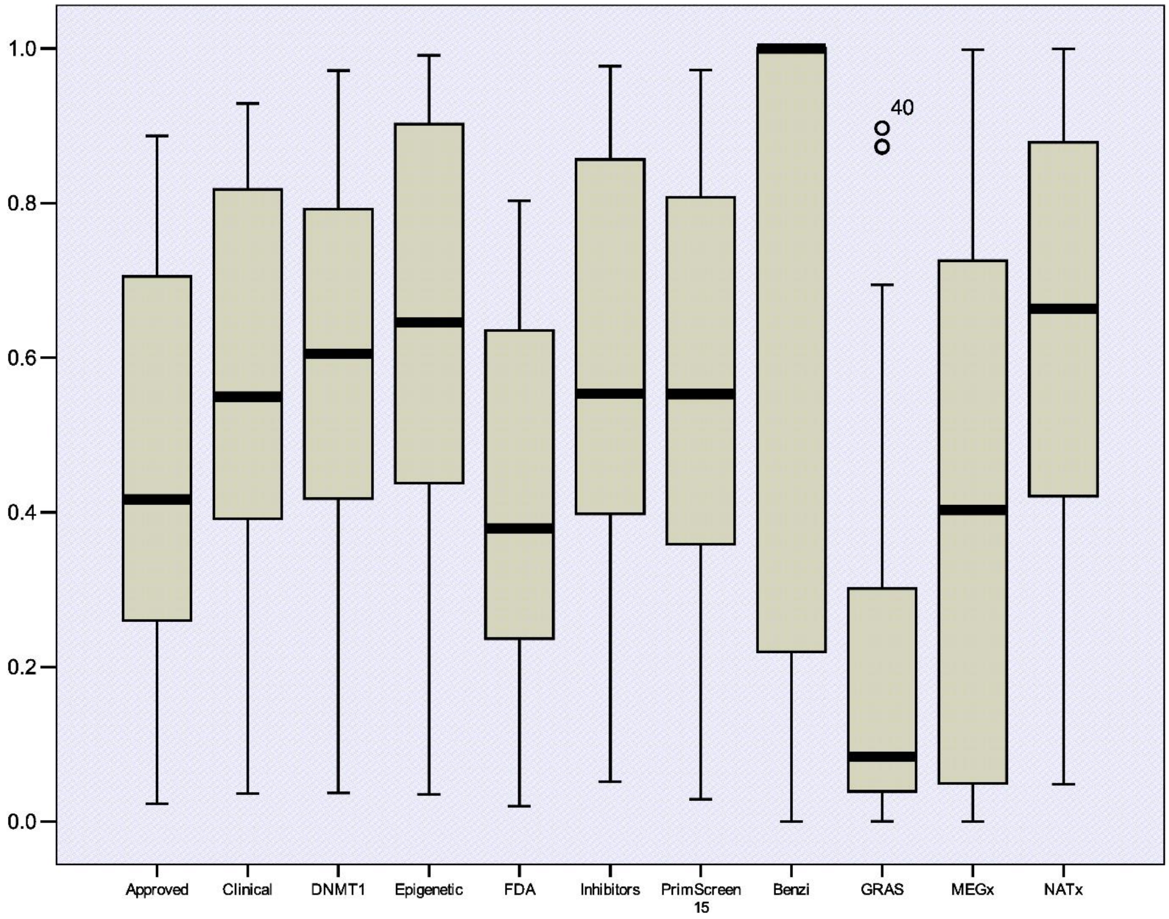

Fig. S7 Probability distribution of the 198 significant bit positions recovered from the original databases represented by PubChem fingerprint at threshold of 0.6.

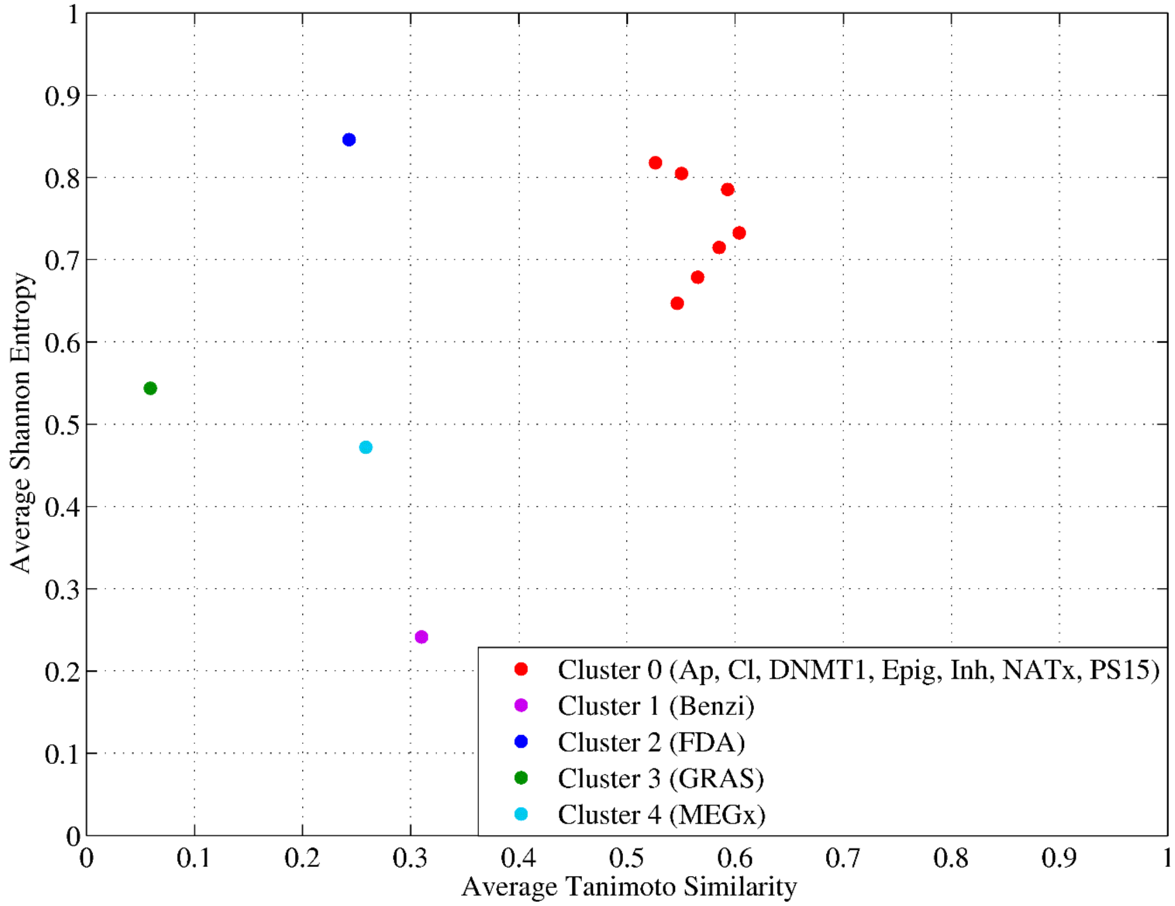

Fig. S8 Relationship Shannon Entropy and DFP/Tanimoto similarity and k-mean Euclidean clustering for the ten compound data sets in Table 2 at threshold of 0.7.

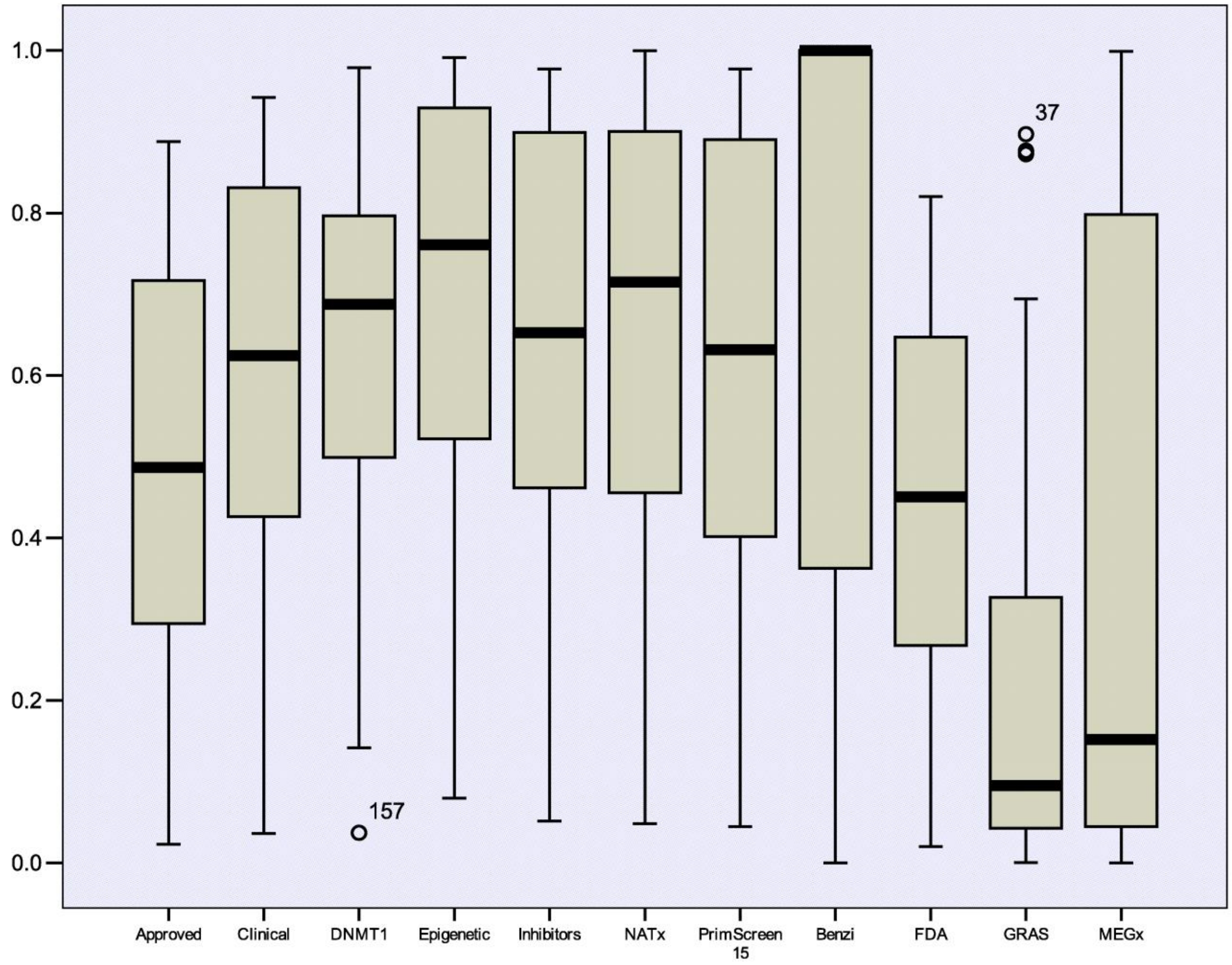

Fig. S9 Probability distribution of the 198 significant bit positions recovered from the original databases represented by PubChem fingerprint at threshold of 0.7.
